# Essential multimeric enzymes in kinetoplastid parasites: A host of potentially druggable protein-protein interactions

**DOI:** 10.1371/journal.pntd.0005720

**Published:** 2017-06-29

**Authors:** Leah M. Wachsmuth, Meredith G. Johnson, Jason Gavenonis

**Affiliations:** Department of Chemistry, Dickinson College, Carlisle, Pennsylvania, United States of America; McGill University, CANADA

## Abstract

Parasitic diseases caused by kinetoplastid parasites of the genera *Trypanosoma* and *Leishmania* are an urgent public health crisis in the developing world. These closely related species possess a number of multimeric enzymes in highly conserved pathways involved in vital functions, such as redox homeostasis and nucleotide synthesis. Computational alanine scanning of these protein-protein interfaces has revealed a host of potentially ligandable sites on several established and emerging anti-parasitic drug targets. Analysis of interfaces with multiple clustered hotspots has suggested several potentially inhibitable protein-protein interactions that may have been overlooked by previous large-scale analyses focusing solely on secondary structure. These protein-protein interactions provide a promising lead for the development of new peptide and macrocycle inhibitors of these enzymes.

## Author summary

Neglected tropical diseases caused by parasites of the genera *Trypanosoma* and *Leishmania* affect millions of people, primarily in the developing world. Due to a historical lack of incentive or interest, few new drugs have been developed to treat these conditions. Numerous efforts have targeted the metabolism of trypanothione, an essential molecule for maintaining the redox homeostasis of these parasites. This study uses freely available structural databases and computational tools to identify new druggable sites on several essential proteins in these organisms by disrupting the protein-protein interactions that allow multimeric enzymes to function. Five of the targets identified in this study are involved in redox homeostasis, while the remainder are involved in other essential metabolic or biosynthetic processes. Nine have been identified in other computational databases, and two have already been experimentally verified, which suggests that protein-protein interaction inhibition of multimeric enzymes may be a general and viable route for the development of new trypanocidal agents.

## Introduction

Infections caused by the kinetoplastid parasites *Leishmania spp*., *Trypanosoma brucei*, and *Trypanosoma cruzi* are estimated collectively to put at risk one billion people, resulting in tens of millions of infections and upwards of ten thousand deaths per year [1]. Neglected tropical diseases (NTDs) caused by these parasites primarily occur in the developing world and are infrequently the target of commercial drug-development efforts [2]. A number of highly conserved enzymes are present across these pathogenic species, despite substantial genomic diversity [3]. Furthermore, the proliferation of high-resolution crystallographic data affords the opportunity to identify new mechanisms for inhibiting both established and emerging drug targets in these organisms. Recent drug-repurposing efforts have allowed for the development of promising new leads based on previous work on homologous targets, such as kinases and heat-shock proteins, in human diseases [4,5].

Just as neglected tropical diseases have received comparatively little attention from the drug discovery community, so too have protein-protein interactions (PPIs), which are characterized by larger surface area and lower binding affinity than is typical for drug-like molecules [6,7]. A substantial fraction of the protein-protein interaction energy is localized in a few amino acid residues, known as “hot spots,” which are often surface-exposed hydrophobic amino acid residues [8]. Computational alanine scanning can generally predict these interface hot spots with a 79% success rate [9]. This has led to the successful development of several inhibitors of PPIs [10–12]. Of greatest relevance to NTDs, this approach has been applied to inhibition of the cysteine protease cruzain, based on the interaction with its native inhibitor chagasin [13]. Targeting PPIs of multimeric enzymes [14,15] in these pathogens, by avoiding the highly conserved substrate-binding domains, should allow for fine-tuning selectivity to avoid inhibition of the homologous host enzymes [15]. This approach has been successful in PPI-based inhibition of the homodimeric enzyme, triosephosphate isomerase (TIM), in *P*. *falciparum* [14] and *T*. *cruzi* [16]. Thus, a systematic analysis of these overlooked targets for neglected diseases may reveal both new drug targets and new approaches to inhibit well-established targets.

## Methods

Structures of multi-protein complexes from the family Trypanosomatidae were obtained using the advanced search functionality of the Protein Data Bank [17]. Structures with >4 Å resolution or >90% similarity were excluded. The PDB files were cleaned to remove headers, retaining only ATOM line entries, using a shell script. Computational alanine scanning [9] was performed using Rosetta 3.6 and PyRosetta [18], with a modified version of the alanine-scanning script originally developed by the Gray lab [19]. The updated Talaris2013 scorefunction [20] was parameterized to match an established general protocol [9,21] without environment-dependent hydrogen bonding terms. Default score function weights were retained, but line 129 of the script was replaced as follows to implement these changes:

scorefxn = create_score_function(‘talaris2013’)

Interfaces that were determined to have at least three hot spots (ΔΔG ≥ 1.0 Rosetta Energy Units (REU), average of 20 scans, 8.0 Å interface cutoff) by this method were further examined for proximity of the hot spots in both primary [22] sequence and secondary/tertiary structure. Complexes with at least two hot spots in close proximity were cross-checked for presence in existing databases of helix [23,24] and loop [25,26] interaction motifs, then with existing literature for experimentally verified interface hot spots, and finally for identity as an established or emerging drug target [27–31]. Amino acid residues falling just below the threshold (ΔΔG between 0.8 and 1.0 REU) were also considered when proximal to multiple interface hot spots. During the preparation of this manuscript, the authors became aware of the Peptiderive server [32], which allows for the rapid examination of single PPI interfaces for hot-spot rich segments of a defined length. The PPIs identified in this study were subsequently re-examined using Peptiderive to locate decameric “hot segments” for comparison.

## Results and discussion

### Identification and characterization of PPIs

Of the 1,076 kinetoplastid protein structures deposited in the PDB, 207 are multi-chain biological assemblies. Computational alanine scanning identifies 56 structures containing at least three putative interface hot spots (27%). Hot spots are defined as any amino acid residue that, when mutated to alanine, increased the ΔΔG_complex_ by at least 1.0 REU [33], a threshold that generally has a 79% correspondence with experimentally observed hot spots [9]. Among these 56 structures, 46 contain multiple hot spots on the same helix (27) or loop (19). Despite the 90% sequence identity cutoff, several homologous proteins from *Leishmania spp*., *Trypanosoma spp*., and the non-pathogenic model organism *Crithidia fasciculata* appear multiple times (*vide infra*), reducing the number of unique interfaces to 34. Analysis of these 34 complexes reveals 12 unique PPIs that are either established drug targets in *T*. *cruzi*, *T*. *brucei*, or *Leishmania spp*. or essential enzymes and structurally or functionally obligate multimers. Drug targets with inhibitable PPIs, their potentially inhibitory peptide sequences, and a comparison to HippDB, Loopfinder, Peptiderive, and experimental results are listed in Table 1.

**Table 1:** Potential self-inhibitory peptides from multimeric enzymes in kinetoplastid parasites.

Target	Species	Peptide	Helix	Loop	Segment	Assay
**TryR**	*Cf*	71-TIRESAGFGWELD	+	++	++	+ [34]
**GS**	*Tb*	2-VLKLLLEL	+	+	+	
**TXNPx**	*Lm*	145-NDMPVGR	+	++	++	
**G6PDH**	*Tc*	441-AMYLKLTAKTPGLLNDTHQTEL	+	+	+	++ [35]
**6PGDH**	*Tb*	251-LTEHVMDRI	+	+	+	
**RpiB**	*Tc*	141-RIEKIRAIEASH	++	+	++	
**GalE**	*Tb*	111-PLKYYDNNVVGILRLL	++	++	++	
**FPPS**	*Tb*	25-FDMDPNRVRYL	+	+	++	
**TAT**	*Tc*	54-AQIKKLKEAIDS	+	--	++	
**PTR1**	*Lm*	192-TIYTMAKGALEGLTRSAALELA	++	+	++	
**dUTPase**	*Lm*	51-ELLDSYPWKWWK	+	--	++	
**DHODH**	*Lm*	201-VIDAETESVVIKPKQGFG	--	++	++	

Targets for the 12 potentially self-inhibitory peptides identified in this study, organism in which they are found, peptide sequences, and comparison to previous approaches relying on helical secondary structure (HippDB, “Helix”) or loops amenable to cyclization (Loopfinder, “Loop”), identification of decameric hot segments (Peptiderive “Segment”), and experimentally validated *in vitro* enzyme inhibitors (“Assay”). Full sequences and scores for peptides identified in HippDB, Loopfinder, and using the Peptiderive Server can be found in S1 Table. *Cf*: *Crithidia fasciculata*; *Tb*: *Trypanosoma brucei*; *Tc*: *Trypanosoma cruzi*; *Lm*: *Leishmania major*.

+ non-overlapping peptide sequence

++ overlapping sequence

--not found in database

### Trypanothione metabolism and the pentose phosphate pathway

Five targets are involved in the redox metabolism of trypanothione, an essential pathway for the parasites’ antioxidant defense that has been the target of numerous drug development efforts [36]. Central to this pathway is Trypanothione reductase (TryR), an essential enzyme which maintains trypanothione, T(SH)_2_, in the reduced state. T(SH)_2_ is produced from glutathione (GSH), which is both synthesized *de novo* by glutathione synthetase (GS) and scavenged extracellularly from the host. TryR utilizes NADPH as a reductant, produced primarily from the pentose phosphate pathway (PPP) by glucose 6-phosphate dehydrogenase (G6PDH), which itself is induced by the presence of hydrogen peroxide, and 6-phosphogluconate dehydrogenase (6PGDH) [37]. Several enzymes use T(SH)_2_ to detoxify specific reactive oxygen species, including tryparedoxin peroxidase (TXNPx), which reduces hydroperoxides produced by the host’s immune response.

**Fig 1:**
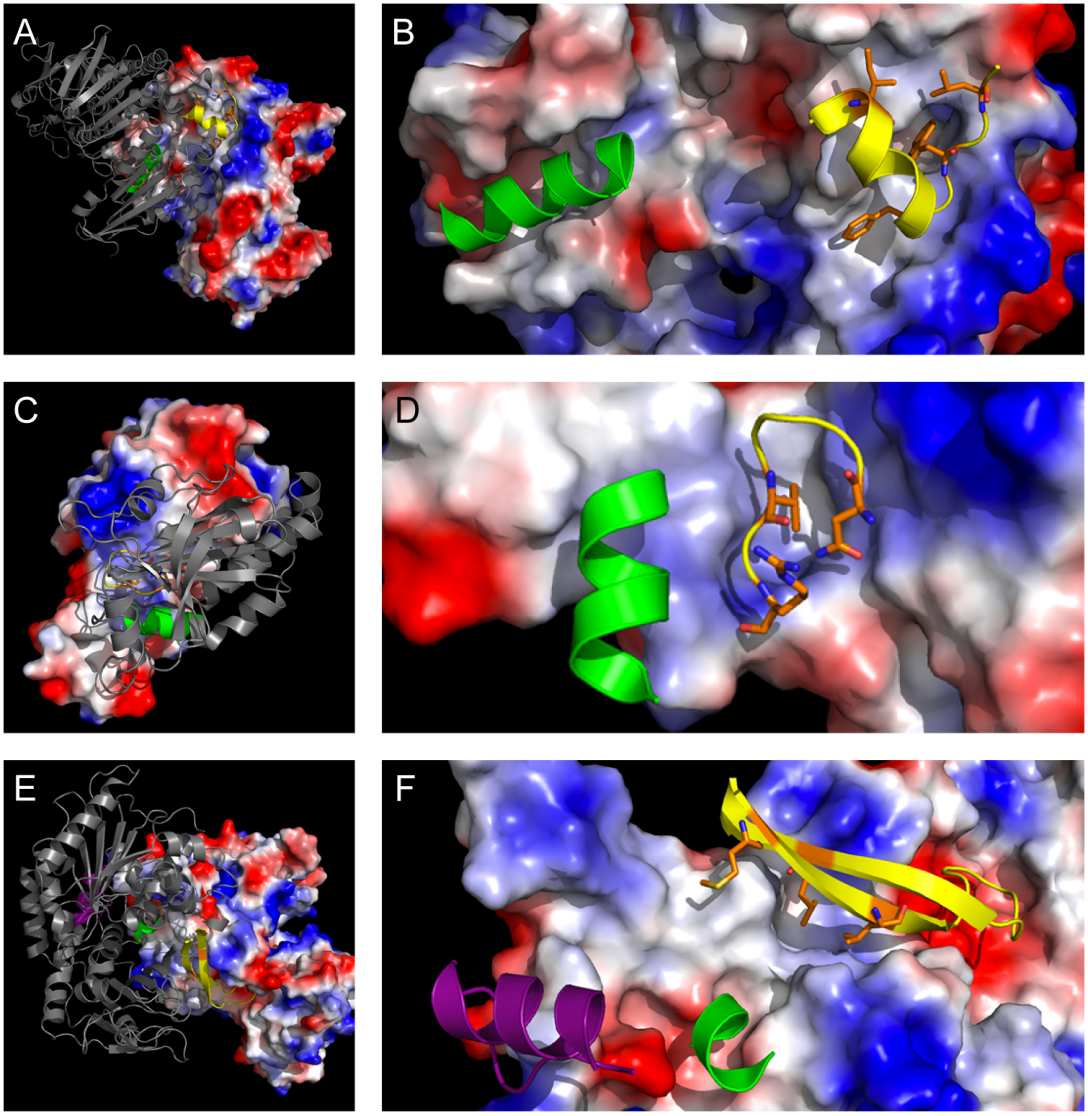
Protein-protein interactions of drug targets involved in redox homeostasis. (A) TryR dimer from *C*. *fasciculata* (PDB: 1FEC) with known inhibitory helix (green cartoon) and predicted inhibitory helix-terminating loop (yellow cartoon) against calculated electrostatic surface. (B) Detail of interface peptide 71-TIRESAGFGWELD containing hot spots Ile-72, Phe-78, Trp-80, and Leu-82 (orange sticks). (C) TXNPx dimer from *L*. *major* (PDB: 4K1F) with predicted helix from HippDB (green cartoon) and predicted inhibitory loop (yellow cartoon) against calculated electrostatic surface. (D) Detail of interface peptide 145-NDMPVGR containing hot spots Asn-145, Val-149, and Arg-151 (orange sticks). (E) G6PDH dimer from *T*. *cruzi* (PDB: 4E9I) with predicted helix from HippDB (green cartoon), helix-turn predicted by Loopfinder (purple cartoon), and long, beta-sheet-anchored loop (yellow cartoon) matching the homologous region of a known inhibitor of *H*. *sapiens* G6PDH. (F) Detail of interface peptide 441-AMYLKLTAKTPGLLNDTHQTEL containing hot spots Met-442, Leu-446, and Leu-462 (orange sticks) clustered in a hydrophobic pocket. Images were rendered using PyMOL v0.99rc6 [42].

Trypanothione reductase is a well-established drug target, with almost all known inhibitors targeting the active site through covalent inactivation of the catalytic cysteine residues or binding of polycationic species in the active site [2,30,38,39]. Recent computational and experimental studies have identified a hot-spot-containing helix in *L*. *infantum* TryR that inhibits TS_2_ reduction by disrupting dimerization of the enzyme, as demonstrated by kinetics and ELISA [34,40]. This helix overlaps, but shares little sequence homology with, a helix that disrupts dimerization of human glutathione reductase (hGR), although it presents a strikingly similar helical face (S1 Fig). The hGR peptide prevents refolding of denatured hGR, but does not inhibit the activity of the native enzyme [41], minimizing the possibility of hGR inhibition from an isosteric TryR inhibitor. Beyond this known mode of inhibition, this study identified a short helix-breaking loop in *C*. *fasciculata* (Fig 1A), *T*. *brucei*, *T*. *cruzi*, and *L*. *infantum* TryR containing three hotspots, Ile-72, Phe-78, and Leu-82, which matches a loop in *Li*TryR identified by Loopfinder (heat score: 3), overlaps a segment predicted by Peptiderive (22% interface energy), and contains one hotspot (Trp-80) that has been verified experimentally (Fig 1) [34]. This proposed inhibitory peptide is predicted to contribute more to the interface energy than the established helix-based inhibitor.

Glutathione synthetase is the second step in *de novo* synthesis of TS_2_, producing glutathione from γ-glutamylcysteine, glycine, and ATP. Knockout of GS in *T*. *brucei* results in a growth-restriction phenotype that is not rescued by addition of exogenous glutathione, suggesting that GS may be druggable [43]. Initial structural characterization of *T*. *brucei* GS had suggested that the high homology in the regions involved in substrate and cofactor binding and catalysis would make GS a suboptimal drug target [44]. However, the helix identified in this study differs greatly from the human homolog in both primary sequence and secondary structure. While the proposed inhibitory sequence, 2-VLKLLLEL, contains only two putative hot spots, Leu-3 and Leu-7, recombinant *Tb*GS with an N-terminal His_6_ tag has drastically reduced catalytic turnover [45], suggesting the importance of the N-terminus in dimerization and providing a plausible route to selective PPI-based inhibition of *Tb*GS in this region. Both HippDB (17%) and Peptiderive (22%) identified segments near the N-terminus predicted to contribute substantially to the stability of the PPI.

Tryparedoxin peroxidase catalyzes the TS_2_-dependant detoxification of peroxides, is an essential enzyme in *T*. *brucei* and *L*. *major*, and has been proposed as a potential drug target due to the constitutively high levels of peroxide, especially in *T*. *brucei* [46–50]. TXNPx is an obligate homodimer, with catalytic cysteine residues for a single active site located on both subunits [48]. This study identifies an unstructured loop containing three hot spots, Asn-145, Val-149, and Arg-151, in *L*. *major*, *T*. *brucei*, and *C*. *fasciculata* (Ile-149) in a hydrophobic cleft on the surface. This peptide matches a loop identified by Loopfinder (heat score: 9) and a segment identified by Peptiderive (27% interface energy). HippDB contains one short, two-turn helix, predicted to contribute only 9% interface energy (Fig 1).

Glucose-6-phosphate dehydrogenase catalyzes the first reaction in the pentose phosphate pathway, oxidizing glucose-6-phosphate to 6-phosphogluconolactone and producing NADPH required for TS_2_ reduction [37]. G6PDH is both an essential enzyme and a validated drug target [51], in addition to being catalytically active in both dimeric and tetrameric forms. Inhibition by peptide-based PPI disruption has been successfully applied to human G6PDH [35]. This study identifies a homologous loop from *T*. *cruzi*, 441-AMYLKLTAKTPGLLNDTHQTEL, containing three hot spots, Met-442, Leu-446, and Leu-462, which are tightly clustered on adjacent strands of a beta sheet in a hydrophobic cleft (Fig 1). This suggests that this approach may find similar success in trypanosomatids with a carefully designed, smaller macrocyclic peptide. Moreover, Peptiderive identifies a decameric segment predicted to contribute 38% of interface energy, which, when extended to include an adjacent two-turn helix, is expected to contribute >50% of interface energy by Loopfinder (heat score: 4). Considering the successful inhibition of hG6PDH and a second predicted inhibitory peptide, G6PDH presents a logical opportunity to explore PPI-based inhibition.

6-phosphogluconate dehydrogenase catalyzes the third step in the pentose phosphate pathway, converting 6-phosphogluconate to ribulose 5-phosphate and CO_2_. G6PDH and 6PGDH are the primary source of NADPH for the reduction of TS_2_ [37,52,53]. This essential enzyme has a highly conserved sequence identity between *T*. *cruzi*, *T*. *brucei*, and *L*. *major*, yet differs substantially from the human 6PGDH homolog, making it an ideal drug target [54]. Substrate analogs have shown potent inhibition of 6PGDH and trypanocidal activity in the low micromolar range [55]. Substrate binding involves residues from both protomers, suggesting PPI disruption may also be a viable inhibition strategy [56]. This study identifies a loop in *T*. *brucei* 6PGDH, 251-LTEHVMDRI, containing three hot spots, Asp-253, Val-255, and Ile-259. HippDB, Loopfinder, and Peptiderive all identified peptides immediately surrounding a helix, 445-YGQLVSLQRDVFG, predicted to contribute 10–13% of the interface energy.

### Essential enzymes beyond trypanothione metabolism

The remaining seven targets represent a variety of essential metabolic and biosynthetic processes. Two targets emerged in sugar metabolism: ribose-5-phosphate isomerase B (RpiB) in the non-oxidative branch of the PPP and UDP-glucose-4’-epimerase (GalE) in galactose catabolism. Three other targets are involved in varied essential biosynthetic processes: Farnesyl pyrophosphate synthase (FPPS) in the isoprenoid biosynthetic pathway, tyrosine aminotransferase (TAT) in tyrosine catabolism, and pteridine reductase (PTR1) in cofactor biosynthesis. Finally, two essential targets are found in nucleotide synthesis: deoxyuridine triphosphate nucleotidohydrolase (dUTPase) and dihydroorotate dehydrogenase (DHODH).

**Fig 2:**
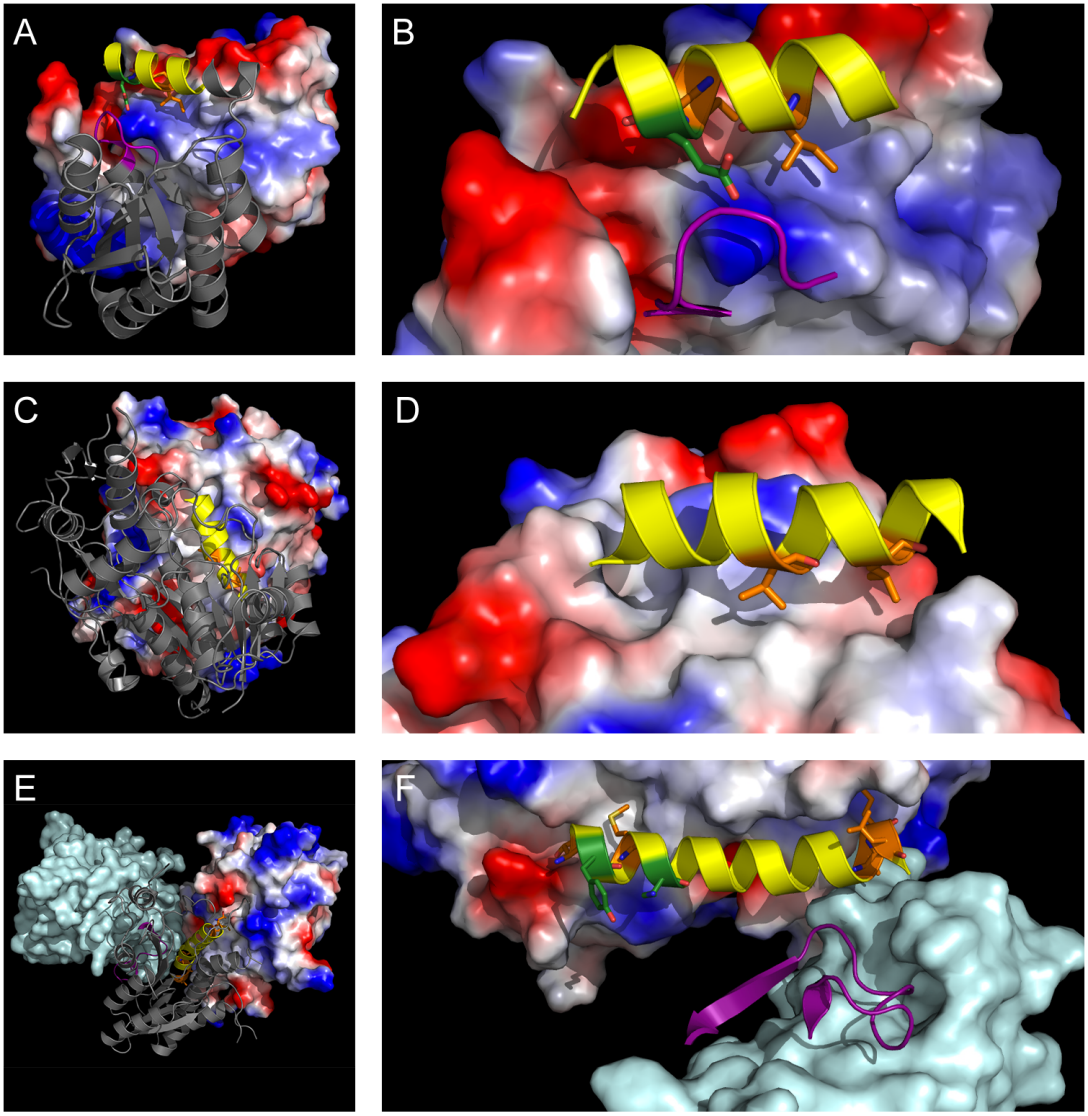
Other essential enzymes where PPI disruption may be a viable inhibition strategy. (A) RpiB dimer from *T*. *cruzi* (PDB: 3M1P) with predicted inhibitory loop (purple cartoon) and helix (yellow cartoon) against calculated electrostatic surface. (B) Detail of peptide 141-RIEKIRAIEASH containing hot spots Ile-145 and Ile-148 (orange sticks) and known inactivating mutant Glu-149 (green sticks). (C) GalE dimer from *T*. *brucei* (PDB: 1GY8) with inhibitory helix (yellow cartoon) predicted by HippDB, Loopfinder, Peptiderive, and this study against calculated electrostatic surface. (D) Detail of peptide 111-PLKYYDNNVVGILRLL containing hot spots Val-119 and Leu-123 (orange sticks) on adjacent turns of the helix. (E) Chains A (grey cartoon), B (electrostatic surface), and C (pale blue surface) of PTR1 tetramer from *L*. *major* (PDB: 2QHX) with predicted A-C interface inhibitory loop (purple cartoon) and A-B interface inhibitory helix (yellow cartoon). (F) Detail of PTR1 helix 5, 192-TIYTMAKGALEGLTRSAALELA, containing predicted hotspots Thr-192, Met-196, Leu-210, Glu-211, and Leu-212 (orange sticks) and catalytic residues Tyr-194 and Lys-198 (green sticks). Images were rendered using PyMOL v0.99rc6 [42].

Ribose 5-phosphate isomerase B catalyzes the interconversion of D-ribose-5-phosphate and D-ribulose-5-phosphate in the non-oxidative branch of the pentose phosphate pathway. RpiB is essential for viability of the bloodstream form of *T*. *brucei* and is a subtype with no mammalian homologue [57–59]. RpiBs are functionally obligate dimers, with catalytic residues spanning both subunits, suggesting that targeting the RpiB interface may a viable inhibition strategy. The peptide sequence from *T*. *cruzi* RpiB identified in this study overlaps a helix predicted by HippDB to contribute 49% of the interface energy. This helix, 140-RRIEKIRAIEASH, contains two predicted hot spots, Ile-145 and Ile-148, on adjacent turns of the helix and the indispensable residue Glu-149. Experimental mutation of this residue disrupts both structure and function in *Ld*RpiB [60]. This helix is also immediately C-terminal to His-138, another deactivating mutant (Fig 2). All four amino acid residues are conserved between *T*. *cruzi* and *L*. *donovani*, suggesting the generality of a peptide helix-based inhibitor.

UDP-galactose-4’-epimerase is an essential enzyme for the growth and survival of trypanosomatid parasites [61]. Unable to acquire galactose from the host, they rely on GalE to synthesize galactose from glucose [61,62]. *T*. *brucei* GalE has 33% homology to the human enzyme [63], and thus has received substantial attention as a target for trypanocidal drugs. Several small-molecule inhibitors have been identified, mainly targeting the active site of the enzyme [62,64,65]. Additionally, GalE is only fully functional as a dimer [61,62], suggesting that the interface of this enzyme is potentially druggable. This study identifies a helix, 111-PLKYYDNNVVGILRLL, with two hotspots, Val-119 and Ile-123, on adjacent turns of the same buried helical face of *T*. *brucei* GalE, overlapping sequences identified by both HippDB (33% interface energy) and Loopfinder (heat score: 9) (Fig 2).

Farnesyl diphosphate synthase, a key enzyme in sterol biosynthesis, catalyzes the sequential condensation of isopentenyl diphosphate and dimethylallyl diphosphate to form geranyl diphosphate and ultimately farnesyl diphosphate, which is the obliged precursor for the biosynthesis of sterols, ubiquinones, dolichols, heme A, and prenylated proteins [66]. Recently, FPPS has been validated as a drug target [67] and the sterol biosynthesis pathway has been targeted at numerous other steps [68]. Most established inhibitors are bisphosphonate substrate mimics; however, they are commonly associated with poor drug-like characteristics [69–72]. FPPS is a functionally obligate homodimer, with the active site located at the protein-protein interface [66,73]. This study identifies a turn between two helices, 25-FDMDPNRVRYL containing three hotspots, Phe-25, Tyr-34, and Leu-35, in *T*. *brucei* FPPS [66]. This same segment is identified by both Peptiderive (15% interface energy) and Loopfinder (heat score: 3).

Tyrosine aminotransferase, which is involved in the first step of amino acid catabolism, catalyzes transamination for both dicarboxylic and aromatic amino-acid substrates [74]. Structural studies suggest that TAT is only fully functional in the dimeric state [75]. TAT is overexpressed in *T*. *cruzi* from patients with acute Chagas [76] and associated with resistance to oxidative damage. This study identifies a three-turn interface helix, 54-AQIKKLKEAIDS, in *T*. *cruzi* and *L*. *infantum* TAT with two proximal hotspots, Leu-59 and Ile-63, on adjacent turns, presenting a hydrophobic face buried in the opposite protomer. This is in contrast to the only helix found in HippDB, 275-PSFLEGLKRVGMLV (15% interface energy), which interacts primarily with the domain-swapped, N-terminal 15 amino acids.

Pteridine reductase, a short-chain reductase, participates in the salvage of pterins, for which trypanosomatids are auxotrophic [77]. PTR1 catalyzes the NADPH-dependent two-stage reduction of oxidized pterins to the active tetrahydro-forms and provides an alternate pathway for folate reduction, allowing *de novo* thymidylate synthesis to occur even in the presence of methotrexate [77,78]. PTR1 is essential in *T*. *brucei* and has been targeted in numerous small-molecule efforts [79–82]. The enzyme is a functional tetramer with substantial surface contacts between the A chain and B and C chains [79,83], suggesting the viability of targeting the PPI. This study identifies six hotspots on helix 5 of *L*. *major* PTR1, with hotspots clustered in hydrophobic pockets at the N-terminal (Thr-192 and Met-196) and C-terminal (Leu-210, Glu-211, Leu-212, and Leu-215) ends of an otherwise convex surface at the A-B interface (Fig 2). The C-terminal portion of this helix is predicted by Peptiderive to contribute 24% of the A-B interface energy and is positioned to mediate the A-C interaction as well. This same helix was identified by HippDB (67% A-B interface energy) and contains two key catalytic residues, Tyr-194 and Lys-198, mutation of which inactivates PTR1 [84]. Loopfinder identified a complementary loop (heat score: 9) that appears to contribute substantially to the A-C interaction.

Deoxyuridine triphosphate nucleotidohydrolase is necessary for both DNA repair and *de novo* synthesis of dTTP. It converts dTUP to dUMP and pyrophosphate. dUTPase maintains a high ratio of dTTP:dUTP, preventing accidental incorporation of uracil into DNA [85,86]. This enzyme was shown to be essential in *L*. *major* and *T*. *brucei* with decreased proliferation in both the procyclic and bloodstream forms of the organism [85,87]. *T*. *cruzi* dUTPase, an obligate dimer, shows little homology to the human counterpart, which is a functional monomer, contributing to its potential as a drug target [86,87]. However, among trypanosomatids, the interface residues are highly conserved [85]. This study identifies an unstructured loop on the interface of *L*. *major* dUTPase, 51-ELLDSYPWKWWK, with two hotspots, Leu-53, Trp-58, in close proximity. An overlapping segment was identified by Peptiderive (35% interface energy). Trp-58, Trp-60, and Trp-61 are buried in a deep hydrophobic cavity on the opposite protomer, although only the former was identified by this computational alanine scan.

Dihydroorotate dehydrogenase catalyzes the oxidation of dihydroorotate to orotate in the *de novo* pyrimidine biosynthetic pathway [88]. The highly conserved DHODHs found in trypanosomatids bear less than 20% sequence homology to the analogous human enzyme [89,90]. DHODH knockout studies demonstrated that the protein is essential in *T*. *cruzi* and an obligate dimer [89,90], suggesting that DHODH would be an ideal drug target in trypanosomatids. This study identifies a long, unstructured loop in *T*. *brucei* DHODH, 202-VIDAETESVVIKPKQGFG, containing three hotspots, Ile-203, Val-210, and Phe-218, tightly clustered in a hydrophobic groove. Both Loopfinder (heat score: 3) and Peptiderive (23% interface energy) identify overlapping portions of this peptide, suggesting an ideal starting point for the development of macrocyclic inhibitors.

### General trends and future outlook

In the past decade, inhibition of PPIs has evolved from the short, primary epitopes exemplified by RGD-peptide-like integrin antagonists and AVPI-peptide-like Smac mimetics to include clinically relevant molecules that recapitulate increasingly complex secondary and tertiary structures like those presented by the BCL family and IL-2, respectively [12,91]. The diversity of topologies, interaction motifs, and binding affinities at these interfaces presents an intriguing challenge for the development of new PPI inhibitors [7]. PPI-based inhibition of NTD targets has achieved some pre-clinical successes, including non-peptide inhibitors of the cysteine protease cruzain [92,93], and interface-peptide-derived inhibitors of triosephosphate isomerase [14,16].

Ultimately, this analysis identified solely homomultimeric enzymes. This is likely due to the bias of existing structural data towards these types of targets, which have received substantial attention as targets for structure-based design of small-molecule inhibitors [28,31,94]. Of the 207 multi-chain trypanosomatid crystal structures in the PDB, 148 (71.5%) are for enzymes (Table 2). Nevertheless, two of the 12 targets identified in this study have been successfully inhibited by interface-derived peptides. An interface-derived peptide helix has been demonstrated to inhibit *Li*TryR through a mechanism that disrupts the PPI [34,40]. This helix had also been identified by HippDB as potentially contributing 15% of the interface energy, while the loop region identified in this analysis is predicted to contribute 22%. Similarly, the G6PDH interface peptide matches a homologous region in the human enzyme, which has been successfully developed into an inhibitory peptide [35]. Given the proximity of the hot spots in space rather than sequence, it appears amenable to inhibition by a macrocycle or peptidomimetic. Overall, PPI-based inhibition of multimeric enzymes [14,15] represents a complementary, but underutilized, approach to these targets.

**Table 2:** Classes of multi-chain kinetoplastid enzymes with structural data in the PDB.

Enzyme Class	Number	Percent
Oxidoreductases	38	18.4%
Transferases	44	21.3%
Hydrolases	23	11.1%
Lyases	18	8.7%
Isomerases	18	8.7%
Ligases	7	3.4%
**Total**	**148**	**71.5%**

The interface peptides identified in this study predominantly contain hot-spot amino acids with aliphatic side chains (Leu, 29%; Ile, 24%; Val, 12%) and Phe (9%). Surprisingly [95–97], other aromatic amino acids (Tyr, 3%; Trp, 3%) appear to be underrepresented in this analysis. These percentages do not differ substantially from the hot spots found over the entire interface (Leu, 30%; Ile, 16%; Val, 16%; Phe, 13%; Tyr, 3%; Trp, 2%). Bogan and Thorn observed a general enrichment of Trp, Tyr, and Arg at interface hot spots [98]; the Loopfinder dataset observed enrichment of Trp, Phe, His, Asp, Tyr, Leu, Glu, and Ile in hot loops [25]. This contrast is most apparent when examining specific PPIs in this study. In the case of TryR (Fig 1), an experimentally verified hot spot (Trp-80) [34] was not identified by the computational alanine scan, despite being buried in the opposite chain of the protein. TryR Trp-80 is conserved across kinetoplastids (S5 Table), as are two of the three calculated hot spots, Ile/Leu-71 and Phe-78. Similarly, the hydrophobic face presented by the interface helix identified for GalE contains a third residue, Tyr-115, not identified in this analysis, but contained in the single alpha-turn found by HippDB as contributing 33% of interface energy (Fig 2). As in the case of TryR, the GalE interface peptide contains three highly conserved hotspots, Pro-111, Val-119, Leu/Ile-123. Sequence conservation of both interface peptides and hot spots was highly variable from protein to protein, with large differences in enzymes such as G6PDH and RpiB, but high homology in TXNPx and PTR1 (S5 Table). Overall, since the interface peptides were identified manually rather than algorithmically, and this is a relatively small data set, it is impractical to extrapolate broader conclusions about the nature of potentially inhibitory interface peptides.

### Conclusion

Computational alanine scanning has revealed 12 drug targets in kinetoplastid parasites that are likely amenable to PPI-based inhibition. While all 12 targets are covered by previous PDB-wide analyses focusing on particular structural motifs, manual inspection of this subset has revealed a number of unique sequences that provide a logical starting point for the development of new inhibitors. Nine of the identified targets have sequences overlapping those identified in previous databases, and two have been experimentally verified, suggesting the potential generality of PPI-based inhibition for these homomultimeric enzymes. Moreover, this approach leverages the power of freely available databases and computational tools, allowing for the rapid analysis of newly disclosed structures for novel modes of inhibition. While a generally predictive model of PPI inhibition has yet to be established, the targets identified in this work present particularly attractive opportunities for the exploration of new modes of inhibition for these targets.

## Supporting information

S1 Fig Sequence and structural alignment of *Li*TryR and hGR inhibitory peptides. Structure alignment of the inhibitory peptide helices for *Li*TryR (435-PEIIQSVGICMKM, shown in purple) and hGR (436-QGLGCDEMLQGFAVAVKMGATKAD, shown in teal) taken from PDB structures 2JK6 and 1GRE. In the assumed binding conformation, *Li*TryR residues E436, Q439, I443, K446, and M447 (purple sticks) present a nearly identical buried helical face to hGR residues E442, Q445, V449, K452, and M453 (teal sticks). Image was rendered using PyMOL v0.99rc6 [42].
(TIF)

S1 Table Full comparison of predicted peptide inhibitor sequences with existing databases and tools. HippDB and Peptiderive scores are both listed as % interface energy contributed by the peptide. Loopfinder heat score is determined using three criteria: A) average amino acid ≥ 0.6 REU; B) ≥ 3 hot spots; C) contributes > 50% interface energy. A heat score of 2, 3, or 4 meets only condition A, B, or C, respectively; a heat score of 9 meets all three criteria.
(XLSX)

S2 Table List of interface hot spots identified for PPIs highlighted in Table 1. (XLSX)

S3 Table All PPIs containing at least three interface hot spots. (XLSX)

S4 Table All multimeric protein interfaces considered in this study. (XLS)

S5 Table Comparison of interface peptide sequences across kinetoplastid species. (XLSX)
