# Supplementary figures and images for: Essential multimeric enzymes in kinetoplastid parasites: A host of potentially druggable protein-protein interactions
Source: PLoS Negl Trop Dis. 2017 Jun 29;11(6):e0005720. doi: 10.1371/journal.pntd.0005720 (PMC5507555; doi:10.1371/journal.pntd.0005720)

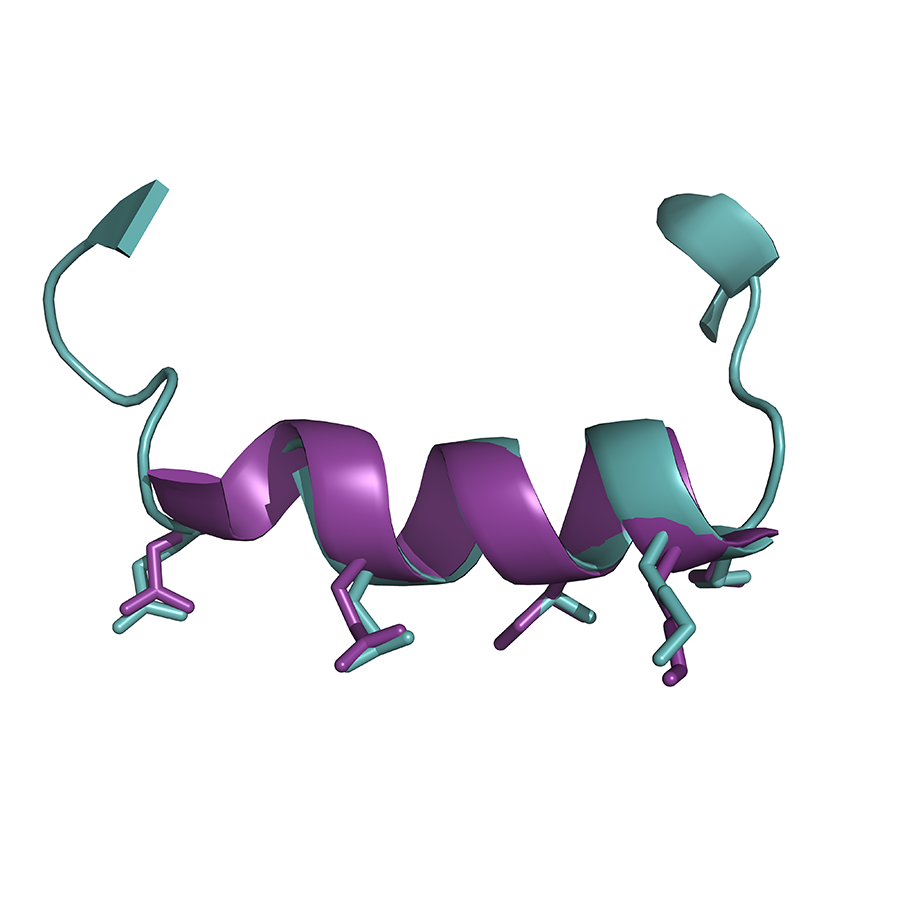

Supplement: S1 Fig — Structure alignment of the inhibitory peptide helices for LiTryR (435-PEIIQSVGICMKM, shown in purple) and hGR (436-QGLGCDEMLQGFAVAVKMGATKAD, shown in teal) taken from PDB structures 2JK6 and 1GRE. In the assumed binding conformation, LiTryR residues E436, Q439, I443, K446, and M447 (purple sticks) present a nearly identical buried helical face to hGR residues E442, Q445, V449, K452, and M453 (teal sticks). Image was rendered using PyMOL v0.99rc6 [42]. (TIF) [file pntd.0005720.s001.tif]
